# Supplementary material for: Templated folding of the RTX domain of the bacterial toxin adenylate cyclase revealed by single molecule force spectroscopy
Source: Nat Commun. 2022 May 19;13:2784. doi: 10.1038/s41467-022-30448-8 (PMC9120197; doi:10.1038/s41467-022-30448-8)
Supplement: Supplementary file 1 — Supplementary Information [file 41467_2022_30448_MOESM1_ESM.pdf]

Templated Folding of the RTX Domain of the Bacterial Toxin Adenylate Cyclase Revealed by Single Molecule Force Spectroscopy

Han Wang[1,2], Guojun Chen[1] and Hongbin Li[1]*
[1]Department of Chemistry
University of British Columbia
Vancouver, BC V6T 1Z1
Canada
[2]Current address: State Key Laboratory of Precision Measuring Technology and Instruments
School of Precision Instrument and Optoelectronics Engineering
Tianjin University
Tianjin 300072
P. R. China

Supplementary Information

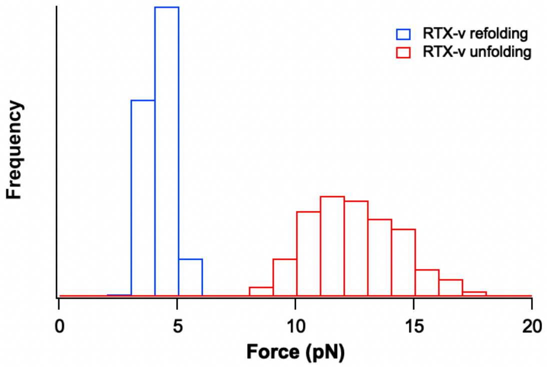

Supplementary Figure 1. Unfolding (n=209) and refolding (n=209) force histograms of RTX-v in RTX-iv-v at a pulling speed of 50 nm/s. For simplicity, the unfolding forces of the two state and three-state unfolding events of RTX-v were grouped together, so are the folding forces.

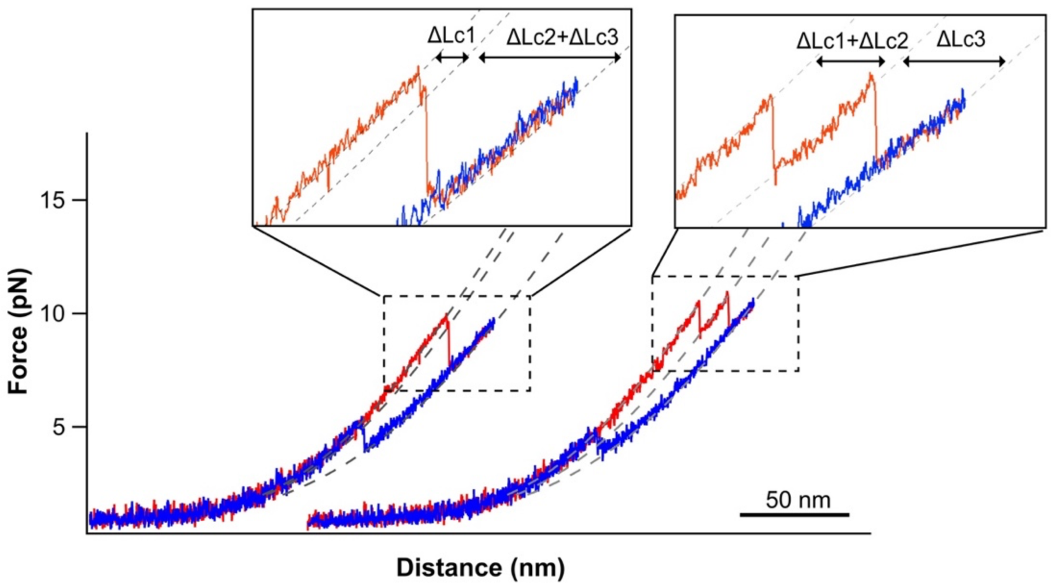

Supplementary Figure 2. Representative *F-D* curves of RTX-iv showing three-state unfolding behaviors. The dashed lines are pseudo WLC fits.

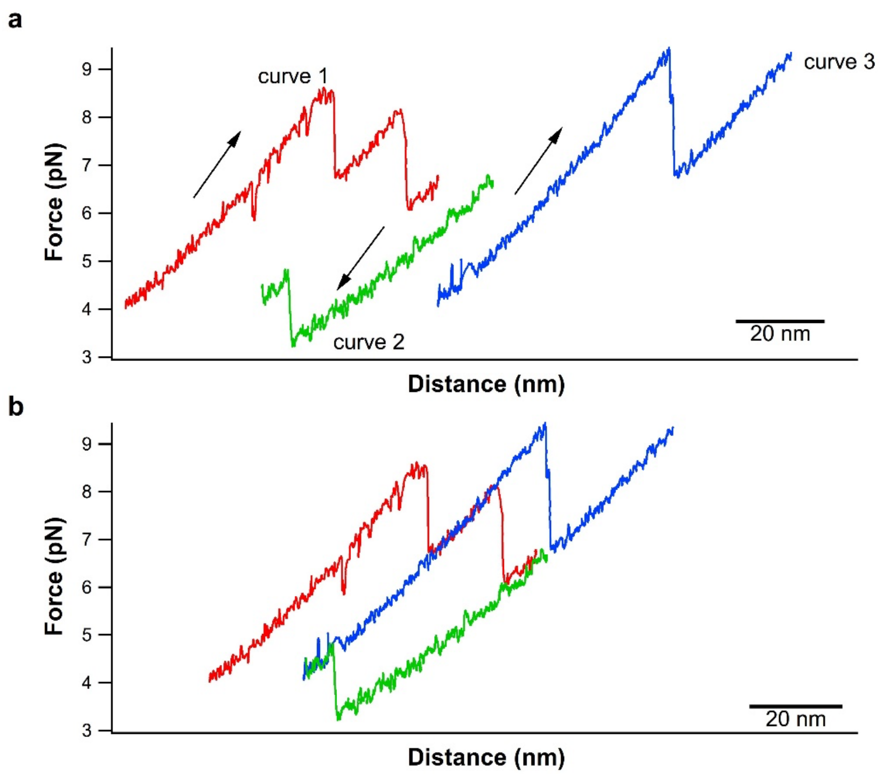

Supplementary Figure 3. Consecutive stretching-relaxation *F-D* curves enable the assignment of the refolding events of RTX-iv and RTX-v. For clarity, three consecutive curves are offset relative to one another horizontally in **a**. In **b**, the three curves are not. In the first stretching curve (curve 1), the unfolding events of RTX-iv and v can be readily assigned based on their ΔLc (40 nm for RTX-iv and 46 nm for RTX-v). The unfolded molecule was relaxed until the first refolding event was observed (curve 2), and then the molecule was stretched again to unfold this refolded RTX block (curve 3). The observed unfolding event in curve 3 matched the RTX-v unfolding event in curve 1. Therefore, the first refolding event can be assigned to the refolding of RTX-v without any ambiguity.

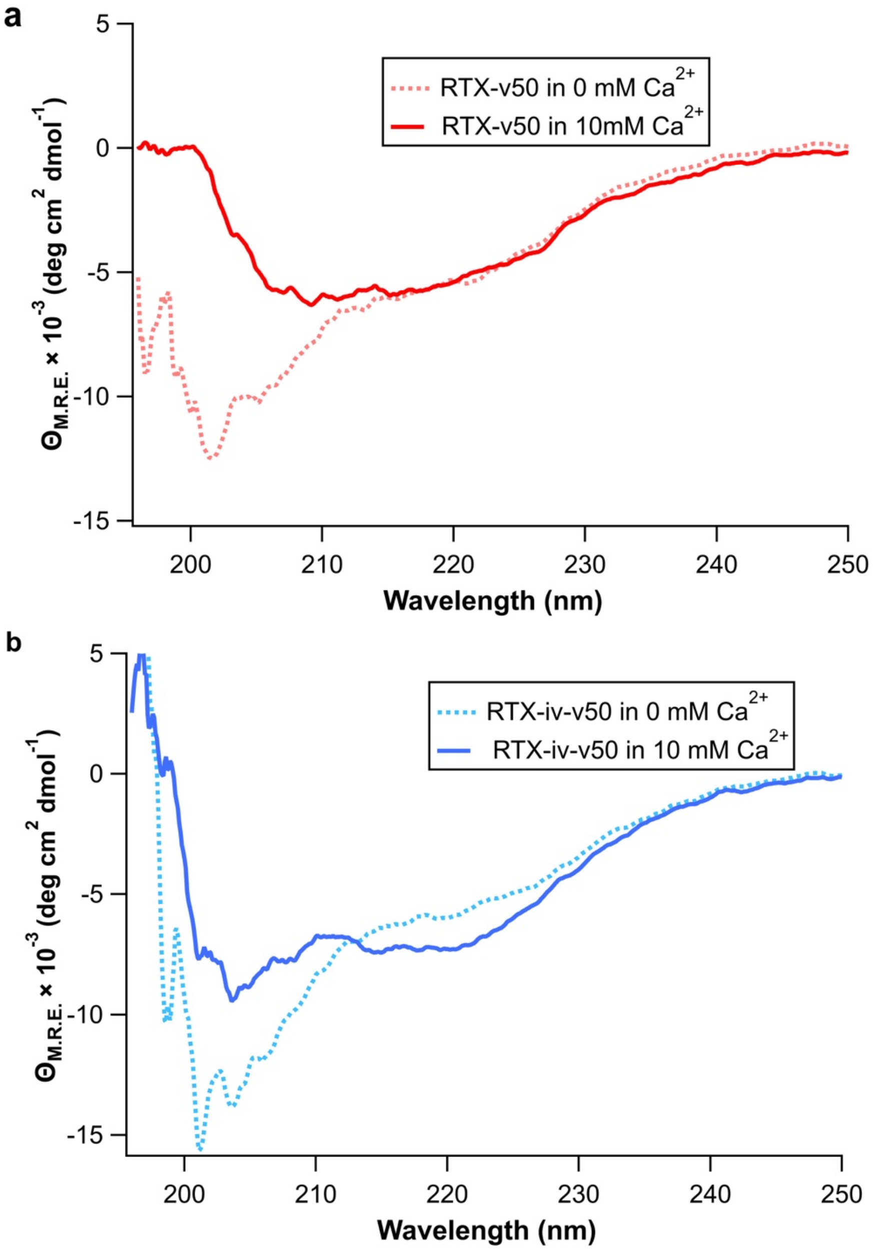

Supplementary Figure 4. Far UV CD spectra of RTX-v_{50} (**a**) and RTX-iv-v_{50} (**b**) in Tris-HCl and Tris-HCl+10 mM Ca^{2+}. After the addition of Ca^{2+} ions, the CD spectra of both constructs showed an obvious shift at ~202 nm, indicating that both apo-RTX-v_{50} and apo-RTX-iv-v_{50} are random coil in the absence of Ca^{2+}, and can fold into their Ca^{2+}-loaded folded structures at the presence of 10 mM Ca^{2+}.

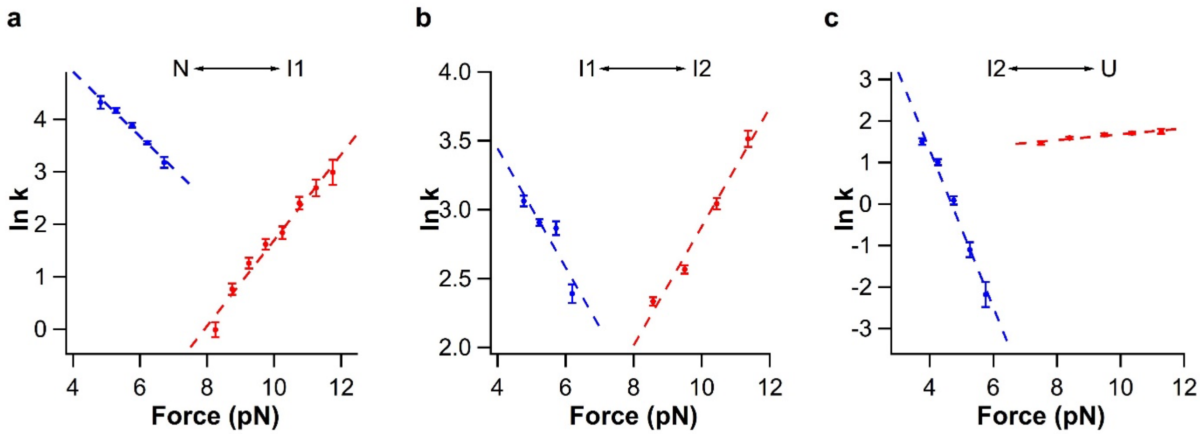

Supplementary Figure 5. Force-dependency of the unfolding (red) and folding (blue) rate constants of RTX-iv for the pathways of **a** N↔I1, **b** I1↔I2 and **c** I2↔U. The rate constants were calculated using the Oesterhelt method by fitting to the dwell-time distribution. Error bars indicate standard deviation of the fitting parameters. Dashed lines correspond to the fits of Bell-Evans model to the experimental data. Kinetic parameters are summarized in Supplementary Table 1. For the unfolding part, equation $\ln k = \ln\alpha_0 + F\Delta x_u$ was used; for folding part, $\ln k = \ln\beta_0 - F\Delta x_f$ was used, where α_0 and β_0 are the intrinsic unfolding and folding rate constant at zero force, Δx_u and Δx_f are the distance between the native state to the transition state and unfolded state to the transition state, respectively.

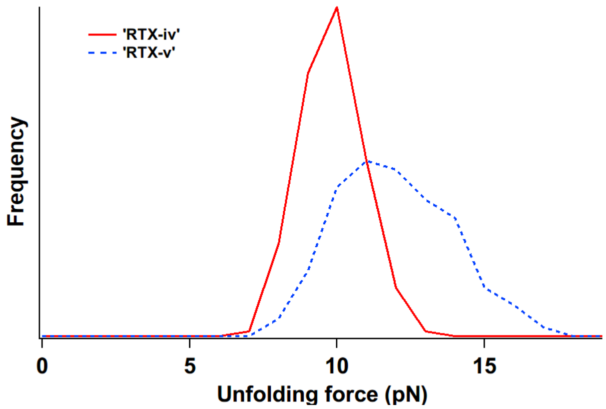

Supplementary Figure 6. Comparison of the unfolding force histograms of RTX-iv (n=209) and RTX-v (n=209) at a pulling speed of 50 nm/s.

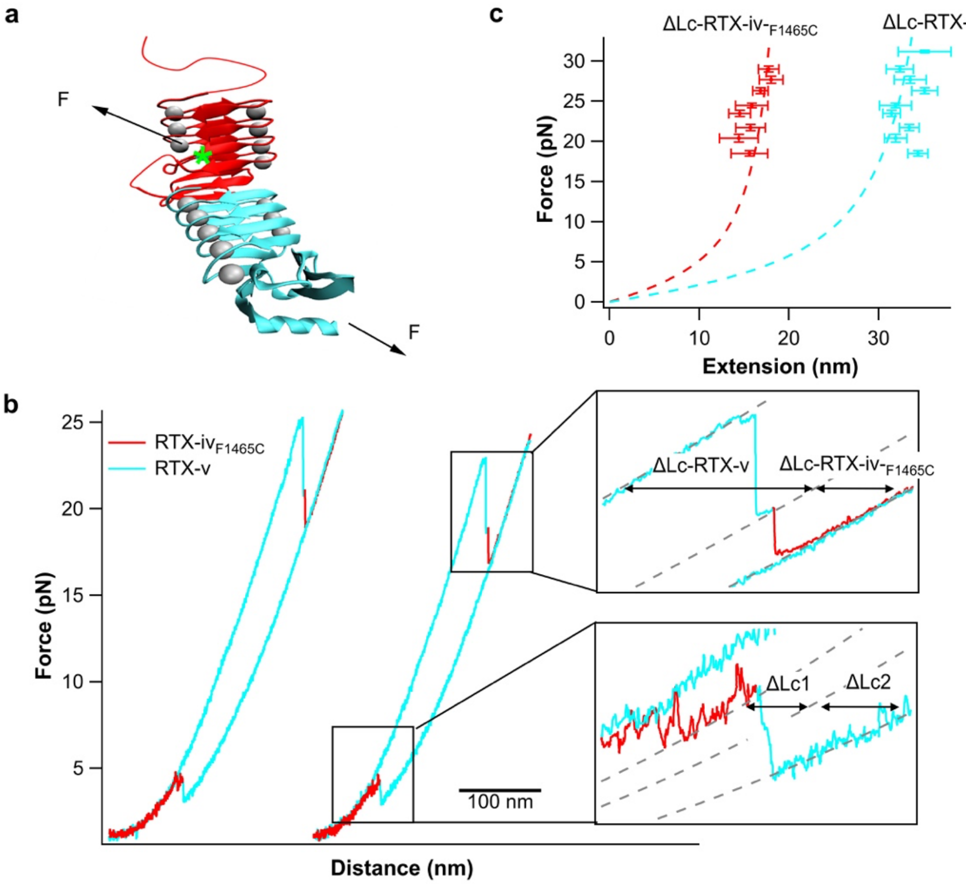

Supplementary Figure 7. Mechanical unfolding/folding of RTX-iv-F1465C-v. **a** Schematics of stretching RTX-iv-F1465C-v from its C-terminus and Phe1465 (indicated by the green asterisk). **b** Representative force-distance curves showing the unfolding/folding of RTX-iv-F1465C -v at a pulling speed of 20 nm/s. The unfolding/folding of RTX-iv-F1465C is colored in red, and the unfolding/folding of RTX-v is colored in cyan. The unfolding and folding both initiate from RTX-v. **c** The WLC fitting of the unfolding of force-extension relationships of the unfolding of RTX-iv-F1465C (red) and RTX-v (cyan), respectively. The WLC fits yielded a persistence length of 0.8 nm and ΔLc (RTX-iv-F1465C) of 22.5 ± 0.4 nm (n=91), and ΔLc (RTX-v) of 44.6 ± 0.9 nm (n=91), respectively, in good agreement with the theoretical calculations. (RTX-iv-F1465C: 64 aa × 0.36 nm/aa – 1.0 nm = 22.0 nm, where 1.0 nm is the distance between F1465 and V1528). The error bars are standard deviations.

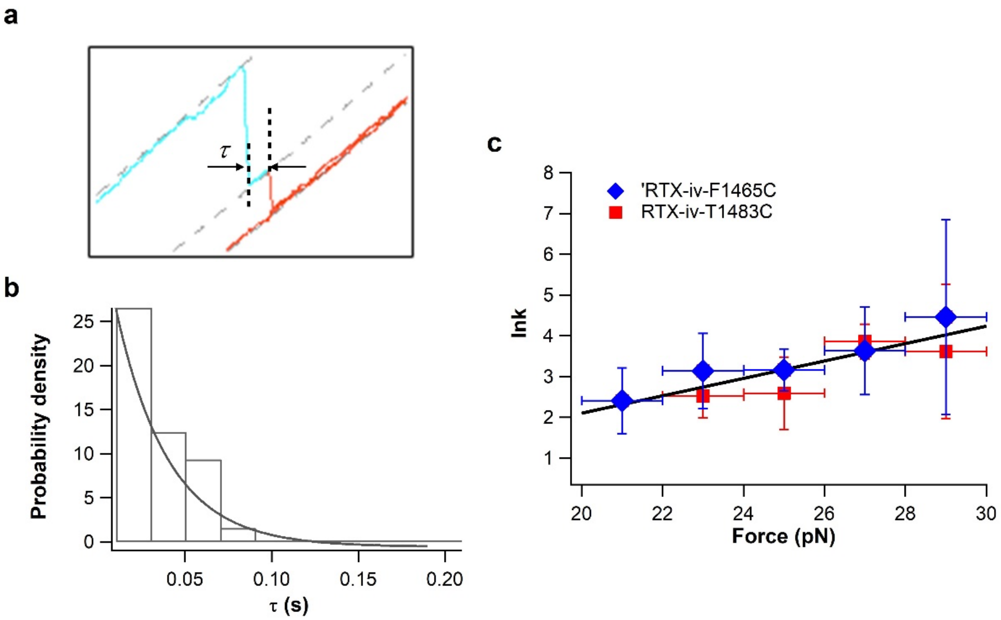

Supplementary Figure 8. Lifetimes of RTX-iv-F1465C and RTX-iv-T1483C. **a** The lifetime τ of RTX-iv can be determined from *F-D* curves of RTX-iv-F1465C-v and RTX-iv-T1483C-v. Due to the short lifetime of RTX-iv, the force acting on the protein was assumed to be constant during its lifetime τ. **b** The lifetime distribution of RTX-iv-T1483C at a stretching force of 27 pN. The solid line is the single exponential fits to the data, which yields the unfolding rate constant k. **c** The plot of *lnk* vs *F* for RTX-iv-F1465C (blue diamond) and RTX-iv-T1483C (red square). For RTX-iv-F1465, the number of events at F= 21, 23, 25, 27, 29 pN are 27, 39, 31, 32 and 25, respectively. For RTX-iv-F1483, the number of events at F= 21, 23, 25, 27, 29 pN are 14, 33, 49, 50 and 21, respectively. The error bars are standard deviations. The solid line is the fit of the combined data to the equation of $lnk = ln\alpha_0 + F\Delta x/(k_BT)$, yielding α_0 of 0.11 ± 0.13 s^{-1} and Δx_u of 0.87 nm.

Supplementary Table 1. Average unfolding and folding forces and kinetic parameters for RTX-iv

	F_u (pN)	F_f (pN)	α_0 (s^{-1})	β_0 (s^{-1})	Δx_u (nm)	Δx_f (nm)
N-I1	9.8 ± 1.1 (n=505)	5.0 ± 0.6 (n=389)	$(1.6 \pm 0.3) \times 10^{-3}$	$(1.6 \pm 0.1) \times 10^{3}$	3.4 ± 0.2	2.5 ± 0.2
I1-I2	9.7 ± 1.0 (n=502)	4.6 ± 0.6 (n=368)	$(2.4 \pm 0.7) \times 10^{-1}$	$(1.8 \pm 0.3) \times 10^{2}$	1.8 ± 0.2	1.8 ± 0.6
I2-U	9.8 ± 1.1 (n=517)	4.1 ± 0.6 (n=469)	2.7 ± 0.3	$(7.1 \pm 0.6) \times 10^{3}$	0.3 ± 0.1	7.8 ± 0.6

The data is presented as average ± standard deviation (S.D.). S.D. of F_u and F_f was calculated directly from the raw data, and S.D. of the rate constants were obtained from the fitting (see Supplementary Fig. 5).
F_u: unfolding force at a pulling speed of 50 nm/s.
F_f: refolding force at a pulling speed of 50 nm/s.
α_0: the intrinsic unfolding rate constant at zero force.
β_0: the intrinsic folding rate constant at zero force.
Δx_u: the distance between the native state to the transition state.
Δx_f: the distance between the unfolded state to the transition state.
